# Characterization of the Dahl salt-sensitive rat as a rodent model of inherited, widespread, persistent pain

**DOI:** 10.1038/s41598-022-24094-9

**Published:** 2022-11-11

**Authors:** Luiz F. Ferrari, Charles Rey, Anna Ramirez, Adam Dziuba, Jacqueline Zickella, Michael Zickella, Hershel Raff, Norman E. Taylor

**Affiliations:** 1 Department of Anesthesiology, University of Utah School of Medicine, 383 Colorow Drive, Salt Lake City, UT 84108 USA; 2 Endocrine Research Laboratory, Aurora St. Luke’s Medical Center, Advocate Aurora Research Institute, Milwaukee, WI 53215 USA; 3 Department of Medicine (Endocrinology and Molecular Medicine), Medical College of Wisconsin, Milwaukee, WI 53226 USA

**Keywords:** Neuroscience, Physiology

## Abstract

Animal models are essential for studying the pathophysiology of chronic pain disorders and as screening tools for new therapies. However, most models available do not reproduce key characteristics of clinical persistent pain. This has limited their ability to accurately predict which new medicines will be clinically effective. Here, we characterize the Dahl salt-sensitive (SS) rat strain as the first rodent model of inherited widespread hyperalgesia. We show that this strain exhibits physiological phenotypes known to contribute to chronic pain, such as neuroinflammation, defective endogenous pain modulation, dysfunctional hypothalamic–pituitary–adrenal axis, increased oxidative stress and immune cell activation. When compared with Sprague Dawley and Brown Norway rats, SS rats have lower nociceptive thresholds due to increased inflammatory mediator concentrations, lower corticosterone levels, and high oxidative stress. Treatment with dexamethasone, the reactive oxygen species scavenger tempol, or the glial inhibitor minocycline attenuated the pain sensitivity in SS rats without affecting the other strains while indomethacin and gabapentin provided less robust pain relief. Moreover, SS rats presented impaired diffuse noxious inhibitory controls and an exacerbated response to the proalgesic mediator PGE_2_, features of generalized pain conditions. These data establish this strain as a novel model of spontaneous, widespread hyperalgesia that can be used to identify biomarkers for chronic pain diagnosis and treatment.

## Introduction

An estimated 10% of the population suffers from debilitating widespread pain each day^1^ at a cost of over $100 billion in health care and lost productivity each year in the United States^2,3^. Chronic widespread pain is a hallmark of several musculoskeletal, neurological, endocrine/metabolic, inflammatory, psychiatric/psychological, and medication-related conditions^4^, and preexisting pain is a risk factor for developing chronic postoperative pain^5^. Although there are FDA-approved medications intended to alleviate generalized pain conditions such as fibromyalgia syndrome (FMS), only 40–61% of patients who take these medications obtain a > 30% pain reduction, leaving a significant number of patients without relief^6–8^. As such, there is a critical need to discover therapies that provide greater symptom relief in a higher percentage of patients.

New drug discovery is typically initiated in animal models to test the safety and efficacy of novel compounds. However, the models currently available do not reproduce key characteristics of clinical widespread pain, which has resulted in a translation gap. Most new therapies that appear to be promising in animal models have failed in human clinical trials^9^. Almost all rodent pain models must be induced using chemical or surgical interventions or by repeated exposure to an environmental stressor^10,11^. Researchers typically perform such interventions in rodent strains such as the Sprague Dawley (SD) rat or C57BL/6 mouse that do not possess genetic susceptibility to pain. This practice limits the applicability of findings to humans, as many pain syndromes arise spontaneously without apparent precipitating factors. Thus, research is hindered by the paucity of animal models that reflect the genetic, environmental, sex-dependent, and psychologic aspects of human chronic widespread pain conditions.

In this study, we characterized the Dahl salt-sensitive rat (SS) as a rodent model of spontaneous persistent hyperalgesia. SS rats were developed to investigate salt-induced hypertension and become profoundly hypertensive when fed a diet containing 2–8% salt^12^. However, on a standard 0.4% sodium diet, such as that used in the current study, SS rats remain normotensive, have glomerular filtration rates indistinguishable from control strains^13^ and do not develop changes in cardiac size or function^14,15^. In the process of studying this strain of rats over many years^16,17^, we observed increased sensitivity to mechanical stimulation before and longer recovery times after surgery when compared to other strains, often showing behaviors compatible with the presence of ongoing pain. As little is known about the neurologic phenotypes of the SS rat, we evaluated this strain for signs of spontaneous hyperalgesia. We compared mechanical nociceptive thresholds and spontaneous pain behaviors with other strains of rats and investigated whether pathophysiological features previously shown to contribute to painful conditions, such as neuroinflammation^18^, deficiencies in the hypothalamic–pituitary–adrenal (HPA) axis^19^, high oxidative stress levels^20^, or impaired central endogenous pain modulation^21^, had an impact on the SS rat nociceptive phenotype. Our findings support the assertion that the Dahl SS rat strain is a novel model of inherited, widespread, persistent pain that can be used as a model for drug development and for studies of putative biomarkers for chronic pain diagnosis and treatment.

## Results

### SS rats have significantly low mechanical nociceptive thresholds

**Figure 1:**
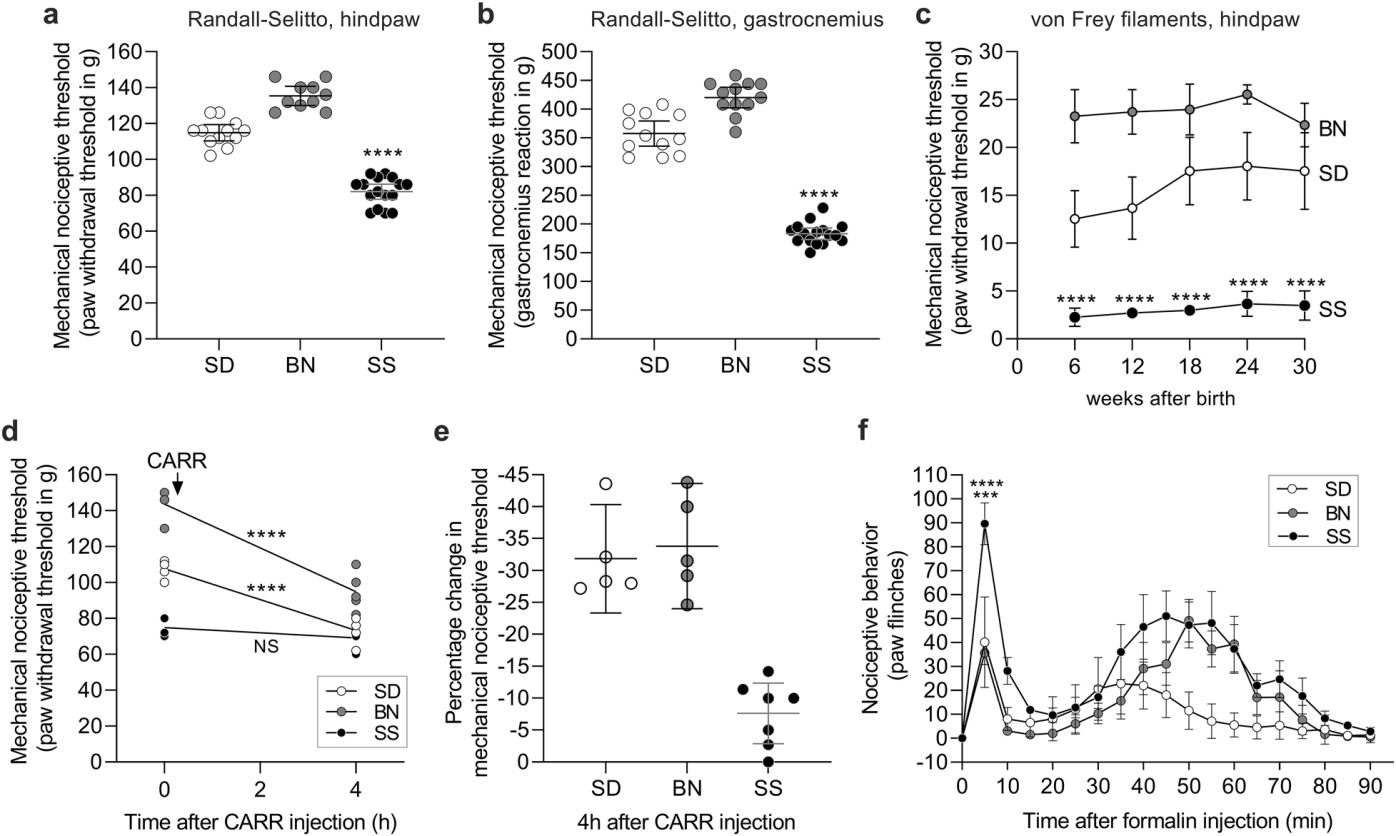
SS rats exhibit higher nociceptive sensitivity. Mechanical nociceptive thresholds of untreated SS (full symbols), SD (empty symbols) and BN (gray symbols) rats were determined on the dorsum of the hindpaw (**a**) and the gastrocnemius muscle (**b**) by the Randall-Selitto test, or in the plantar surface, over the course of 30 weeks, starting at the 6th week post birth, using von Frey filaments (**c**). In all tests, SS rats showed significantly lower nociceptive thresholds than SD and BN rats (panels a and b, respectively: F_(2,35)_ = 164.7 and F_(2,37)_ = 293.2, ****p < 0.0001 for both strains, one-way ANOVA followed by Bonferroni posttest; (**c**): F_(2,29)_ = 171.7, ****p < 0.0001 for both strains, two-way repeated measures ANOVA followed by Bonferroni posttest). (**d**): SS, SD and BN rats received an i.d. injection of CARR (1%) on the dorsum of the hindpaw, and the nociceptive thresholds were evaluated 4 h later. While significant mechanical hyperalgesia (decrease in nociceptive threshold) was observed in the SD (t_4_ = 9.411, *p* = 0.0007, when the thresholds 4 h after CARR were compared to the baseline, paired Student’s t-test) and BN (t_4_ = 8.595, *p* = 0.0010) groups (F_(2,14)_ = 110.0, ****p < 0.0001 when the change in the thresholds induced by CARR in SS rats was compared to those in SD and BN rats, two-way repeated measures ANOVA followed by Bonferroni posttest), no significant change was observed in the SS group (t_6_ = 4.054, *p* = 0.1067, NS). (**e**) shows the percentage change in nociceptive threshold produced by CARR injection (SD = 31.84%; BN = 33.82%; SS = 7.6%), indicating the smaller effect of inflammation on this strain and that its baseline nociceptive threshold is naturally in the hyperalgesic range. (**f**): Formalin (1%) was injected subcutaneously on the dorsum of the hindpaw of SS, SD and BN rats. Nociceptive reaction (paw flinches) to formalin was followed for 90 min. Significant difference in the number of flinches in the SS group was observed in the first 10 min after injection (1st phase), when compared to the other 2 strains (F_(1,10)_ = 24.98, ***p = 0.0005 compared to SD; F_(1,10)_ = 47.91, ****p < 0.0001 when compared to BN group, two-way repeated measures ANOVA followed by Bonferroni posttest). However, the difference in the number of flinches was less robust when the SS rats were compared to the other strains from 15 to 90 min (2nd phase), with no significance compared to BN (F_(1,10)_ = 1.973, *p* = 0.1904), but different from the SD group (F_(1,10)_ = 7.839, *p* = 0.0188). (**a** and **b**): SD, n = 12; BN, n = 10; SS, n = 16. (**c**): SD, n = 12; BN, n = 12; SS, n = 8. (**d** and **e**): SD, n = 5; BN, n = 5; SS, n = 7. (**f**): n = 6 per group.

Paw and gastrocnemius muscle reaction thresholds were evaluated in untreated, naïve SD and Brown Norway (BN) rats and compared to those in normotensive SS rats using the Randall-Selitto device and von Frey filaments. In age-matched rats (12 weeks old), both the hindpaw (Fig. 1a) and the gastrocnemius muscle (Fig. 1b) nociceptive thresholds were markedly lower in the SS group than in the SD and BN groups (*p* < 0.0001 in both skin and muscle, for both strains). This difference was evident as early as 6 weeks of age (Fig. 1c, *p* < 0.0001 when SS rats were compared to SD and BN rats) and was still present when they reached adulthood (30 weeks old, *p* < 0.0001). We compared the SS rat with SD and BN rat strains because (1) the outbred SD strain is used in most pain experiments conducted in rats and (2) the inbred BN strain is the most genetically divergent from the SS, which will allow for significant power in future genetic studies. We also evaluated the response to the inflammogen carrageenan [CARR, injected intradermally (i.d.) on the dorsum of the hindpaw, Fig. 1d and e] using the Randall-Selitto method and observed a robust decrease in the hindpaw mechanical nociceptive threshold in SD (~ 31%) and BN (~ 33%) rats (*p* < 0.0001 for both strains, when the mechanical nociceptive thresholds before and 4 h after CARR injection are compared) down to the pre-injection thresholds of SS rats, providing evidence that the mechanical nociceptive thresholds in SS rats are phenotypically in the hyperalgesic range. In contrast, there was no significant change in the mechanical nociceptive threshold in SS rats in response to CARR (*p* = 0.2838, when the nociceptive thresholds before and at the 4th h post-CARR were compared). For this reason, we will refer to this observation as the SS rat “hyperalgesic phenotype”. To confirm that peripheral sensory neurons in SS rats are physiologically sensitized, i.e., that this strain not only presents lower nociceptive thresholds, but also higher nociceptive responsivity, a separate group underwent formalin testing (Fig. 1f). Formalin (1%) was injected subcutaneously on the dorsum of the hindpaw and the spontaneous nociceptive behavior (paw flinches) was observed over 90 min. When compared to SD and BN rats that received formalin, more robust nociceptive behavior was observed in SS rats (*p* = 0.0005 and *p* < 0.0001, respectively) in the first phase of the test (0–10 min), with a less marked difference between the strains in the second phase (15–90 min, *p* = 0.0291). This finding indicates the higher sensitivity of nociceptors in the SS rats.

### Systemic inflammation plays a role in the Dahl SS hyperalgesic phenotype

**Figure 2:**
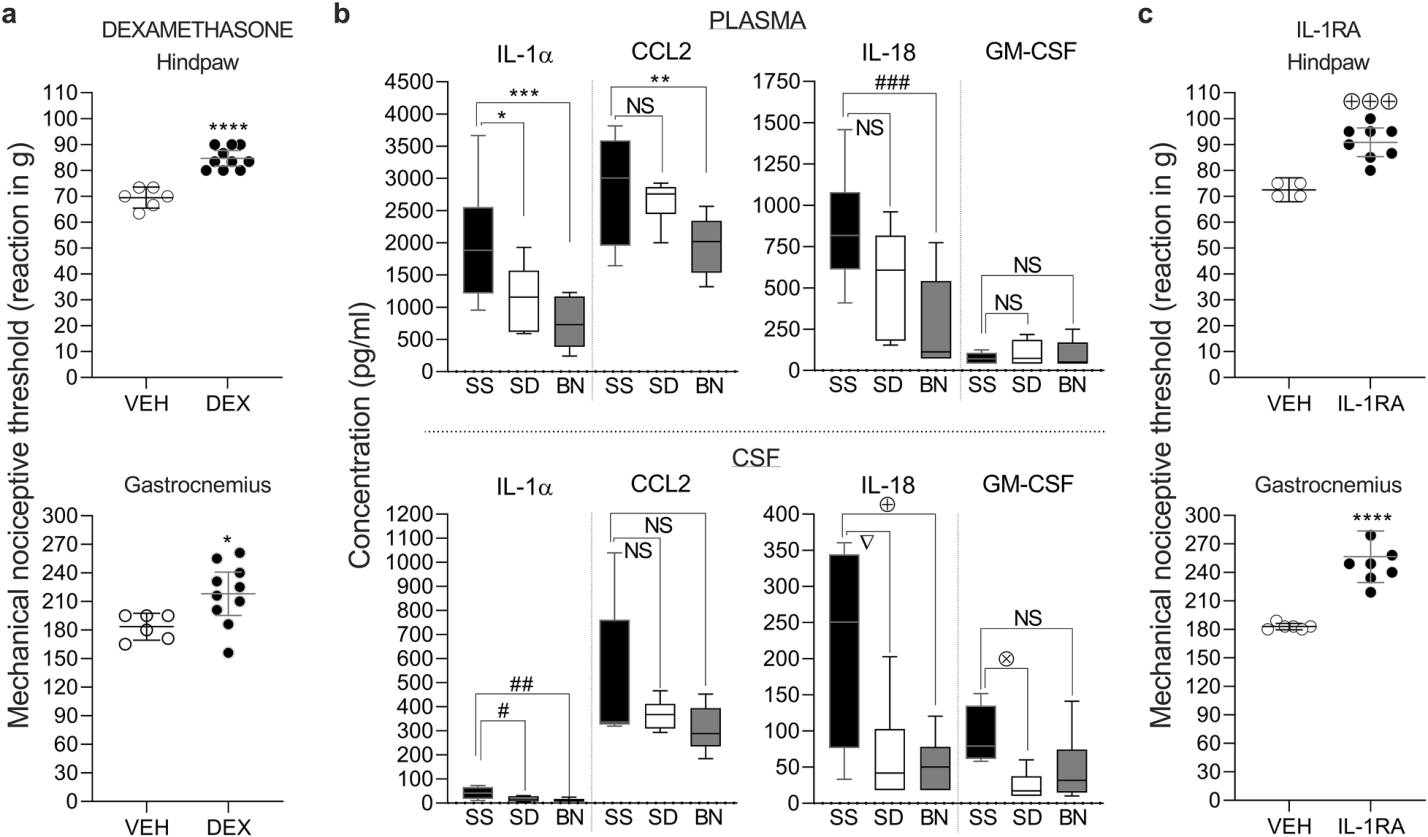
Inflammation contributes to the SS hyperalgesic phenotype. (**a**): SS rats were treated with vehicle (empty symbols) or the glucocorticoid dexamethasone (DEX, 1 mg/kg, s.c., full symbols). Evaluation of the nociceptive thresholds on the dorsum of the hindpaw (upper panel) or the gastrocnemius muscle (lower panel) 24 h later showed a significantly increased threshold in the DEX-treated group (t_14_ = 7.179, ****p < 0.0001 for the hindpaw, compared with the vehicle-treated group, unpaired Student’s t-test; t_14_ = 2.500, *p = 0.0254 for the muscle); (**b**): Multibead immunoassay evaluation of cytokine and chemokine levels in plasma and CSF samples collected from SS (black bars), SD (blank bars) and BN (gray bars) rats. Higher levels of IL-1α were found in the plasma of SS (upper panels, F_(2,22)_ = 9.453, *p* = 0.0011, one-way ANOVA followed by Bonferroni posttest) compared to SD (*p = 0.0439) and BN (***p = 0.0006), as well as in the CSF (lower panels, F_(2,13)_ = 5.492, *p* = 0.0187, with ^#^*p* = 0.0443 for SD and ^##^*p* = 0.0138 for BN rats). IL-18 levels were elevated in the SS plasma (F_(2,23)_ = 9.238, *p* = 0.0011), higher than in BN (^###^*p* = 0.0005), but not different from SD (*p* = 0.1308, NS). In the SS CSF, IL-18 was higher (F_(2,13)_ = 5.722, *p* = 0.0165) than in SD (^∇^*p* = 0.0244) and BN (^⊕^*p* = 0.0161). No difference in GM-CSF concentration was found in the SS plasma (F_(2,22)_ = 0.3469, *p* = 0.7107) compared to SD (*p* = 0.8614, NS) and BN (*p* > 0.9999, NS) or in the CSF of BN (*p* = 0.1821, NS). However, GM-CSF was higher in SS rat CSF (F_(2,13)_ = 3.859, *p* = 0.0483) than in SD rat CSF (^⊗^*p* = 0.0318). Similarly, the CCL2 concentration in SS plasma was higher (F_(2,23)_ = 6.222, *p* = 0.0069) only when compared to BN (**p = 0.0048) but not to those in plasma (*p* > 0.9999, NS) or CSF (*p* = 0.4562, NS) of SD and BN (*p* > 0.1793, NS). (**c**): SS rats received an i.p. injection of IL-1RA (10 mg/kg, full symbols) or vehicle (empty symbols). An increase in nociceptive thresholds in the IL-1RA-treated group was observed 60 min later on the hindpaw (upper panel, t_10_ = 5.200, ^⊕⊕⊕^*p* = 0.0004, compared to the vehicle group, unpaired Student’s t-test) and in the muscle (lower panel, t_12_ = 5.475, ****p < 0.0001). (**a**): vehicle, n = 6, DEX, n = 10; (**b**), plasma: SS, n = 10; SD, n = 6; BN, n = 10; Panel b, CSF: SS, n = 5; SD, n = 6; BN, n = 6; (**c**): vehicle, n = 6, IL-1RA, n = 8.

To investigate whether inflammation plays a role in the SS rat hyperalgesic phenotype, we treated SS rats with the anti-inflammatory glucocorticoid dexamethasone [1 mg/kg, subcutaneously (s.c.)] and evaluated the mechanical nociceptive threshold on the dorsum of the hindpaw (Fig. 2a, upper panel) and on the gastrocnemius muscle (Fig. 2a, lower panel). Systemic injection of dexamethasone produced a significant increase in the nociceptive thresholds in both the hindpaw (*p* < 0.0001, when compared to the vehicle-treated group) and the muscle (*p* = 0.0254) when evaluated 24 h after injection, suggesting that the presence of inflammation is involved in the hyperalgesic phenotype observed in this rat strain. Based on this finding, we next measured cytokine and chemokine levels in the plasma and cerebrospinal fluid (CSF) of SS rats (Fig. 2b) using a multi-bead immunoassay. Significant levels of the cytokines interleukin (IL)-1 alpha (IL-1α) and IL-18 and the chemokines granulocyte–macrophage colony stimulating factor (GM-CSF) and (C–C motif) ligand 2 (CCL2) were detected in SS rats compared to SD and BN rats. These markers have all previously been shown to play a role in inflammatory or chronic pain^22–26^. Higher levels of IL-1α were found in both the plasma (upper panels, *p* = 0.0439 when compared to SD rats; *p* = 0.0006 when compared to BN rats) and the CSF (lower panels, *p* = 0.0443 when compared to SD rats; *p* = 0.0138 when compared to BN rats) of SS rats compared to the other strains. Similarly, the concentration of IL-18 was also higher in SS than in SD (*p* = 0.0244 in the CFS, but not significantly different in the plasma, *p* = 0.1308) and BN (*p* = 0.0005 in the plasma and *p* = 0.0161 for the CSF) rats. The concentrations of GM-CSF in the SS rats were higher than those in the CSF of SD rats (*p* = 0.0318), while CCL2 was comparatively different only in the plasma of BN rats (*p* = 0.0048). The chemokine CXCL1/KC and the cytokines TNFα, IFNγ, IL-1β, IL-6, IL-10, IL-12, and IL-33 were also measured, but the levels were either undetectable or insignificantly different between the strains. Since we found higher levels of IL-1α in the plasma and CSF of SS rats, we investigated the effect of systemic treatment with IL-1RA (10 mg/kg, i.p.), an antagonist of the IL-1 receptor at which IL-1α acts^27^, on the SS hyperalgesic phenotype (Fig. 2c). IL-1RA produced a significant increase in the mechanical nociceptive thresholds in both the hindpaw (Fig. 2c, upper panel, *p* = 0.0004, when compared to the vehicle-treated group) and the gastrocnemius muscle (Fig. 2c, lower panel, *p* = 0.0001) of the SS rats, suggesting a contribution of this cytokine to the SS rat hyperalgesic phenotype.

### Tonically activated glial cells contribute to the low mechanical nociceptive threshold of SS rats

**Figure 3:**
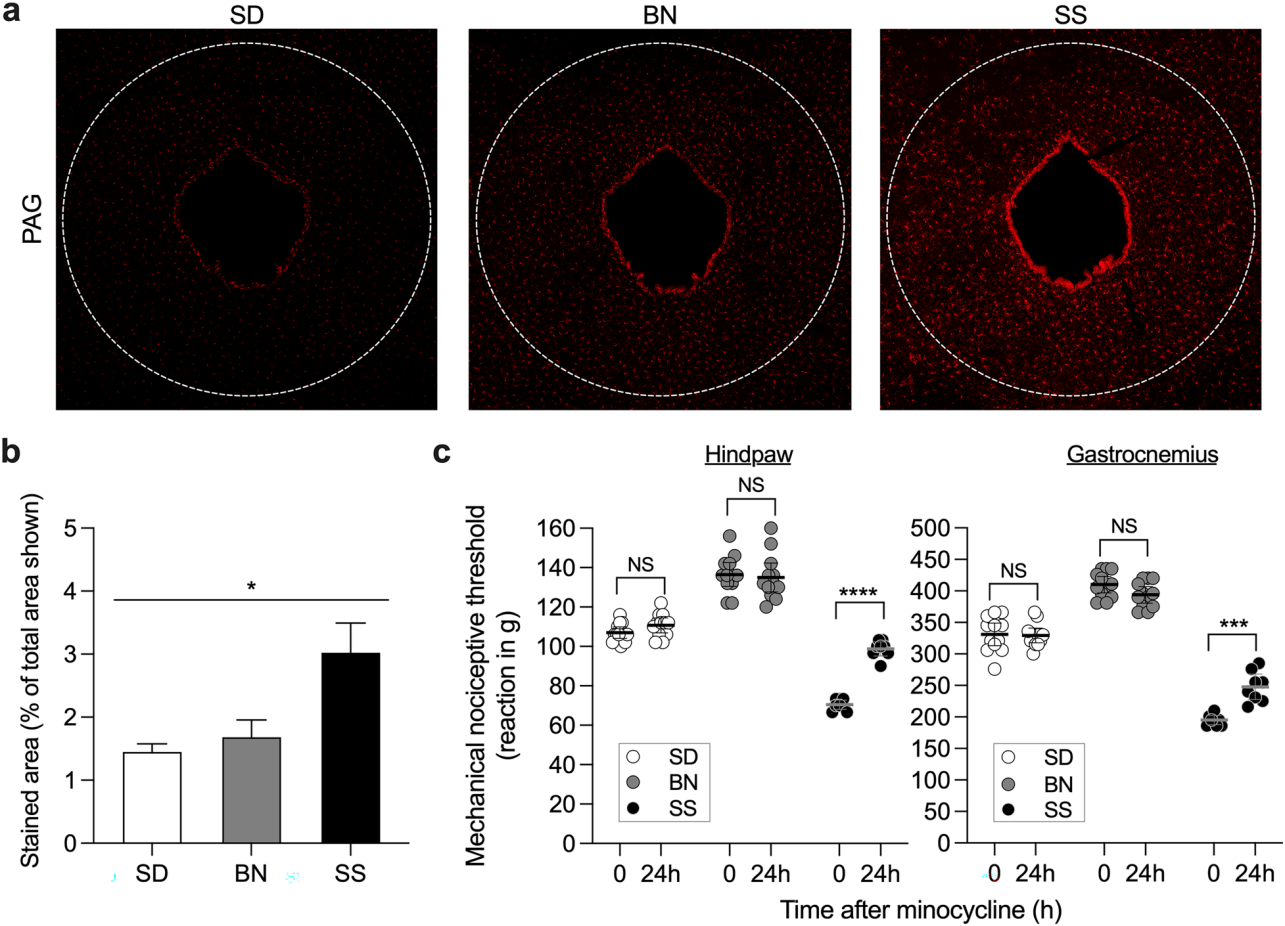
Inhibition of glial cells attenuates the hyperalgesic phenotype in SS rats. (**a**): Naïve SD (left image), BN (center image) and SS (right image) rats had their PAG collected and prepared for immunohistochemistry. Sections were stained with the glial cell activation marker Iba. Quantification of the staining (**b**) showed significant difference in active glial cells across the strains (F_(2,6)_ = 6.883, *p = 0.0280), with higher intensity in sections from SS rats compared to SD (*p* = 0.0277) and BN (*p* = 0.0326) sections (one-way ANOVA followed by Bonferroni posttest). (**c**): SD (open circles), BN (gray circles) and SS (closed circles) rats received an i.p. injection of the glial cell inhibitor minocycline (30 mg/kg). Evaluation of the mechanical nociceptive thresholds, by the Randall-Selitto test, was performed on the dorsum of the hindpaw (left panel) or on the gastrocnemius muscle (right panel), before and 24 h after the injections. We observed a significant difference in the minocycline effect across the groups (F_(2,29)_ = 152.4 for the hindpaw; F_(2,29)_ = 367.8 for the gastrocnemius, *p* < 0.0001 for both when the three groups were compared, two-way repeated measures ANOVA followed by Bonferroni posttest), with increase in the nociceptive threshold in the SS rats (hindpaw: ****p < 0.0001; gastrocnemius: ***p = 0.0001), but no significant change in the SD (hindpaw: *p* = 0.3042; gastrocnemius: *p* > 0.9999, NS) or BN (hindpaw: *p* > 0.9999; gastrocnemius: *p* = 0.2395, NS) groups. Together, these findings suggest a role for glial cells in the SS rat hyperalgesic phenotype. (**c**): SS, n = 8; SD, n = 12; BN, n = 12.

The increased levels of inflammatory mediators and the effect of dexamethasone on the nociceptive threshold suggest a role for systemic inflammation in the SS rat hyperalgesic phenotype. Although there are several possible sources from which cytokines and chemokines can be released, the detection of the microglial-activating chemokine GM-CSF suggests that glial cells might contribute to the SS phenotype. Thus, we investigated whether glial cells were active in SS rats by determining Iba-1 immunoreactivity, a microglia marker, in the periaqueductal grey matter (PAG), an area previously described to play a role in the modulation of nociceptive information^28–31^. We found significantly higher Iba-1 staining in the PAG of SS rats when compared to SD (*p* = 0.0277) or BN (*p* = 0.0326) rats (Fig. 3a and b). Since a number of studies indicate that mediators derived from glial cells contribute to syndromes characterized by widespread pain^18^, we investigated whether their inhibition would impact the low nociceptive threshold observed in the SS rats. We therefore treated SS rats with systemic injection of the glial inhibitor minocycline [30 mg/kg, intraperitoneally (i.p.)] and evaluated the mechanical nociceptive threshold in the hindpaw (Fig. 3c, left panel) and the gastrocnemius muscle (Fig. 3c, right panel) after 24 h compared with BN and SD rats. We saw a significant increase in the nociceptive reaction thresholds (*p* < 0.0001 for hindpaw; *p* = 0.0004 for gastrocnemius, when compared to pre-treatment thresholds) in the SS rats. In contrast, SD and BN groups showed no change in nociceptive threshold (SD: *p* = 0.3042 for hindpaw; *p* > 0.9999 for gastrocnemius; BN: *p* > 0.9999 for hindpaw; *p* = 0.2395 for gastrocnemius). These observations suggest that glial cells play a role in the increased mechanical sensitivity of SS rats.

### Involvement of oxidative stress in the SS hyperalgesic phenotype

**Figure 4:**
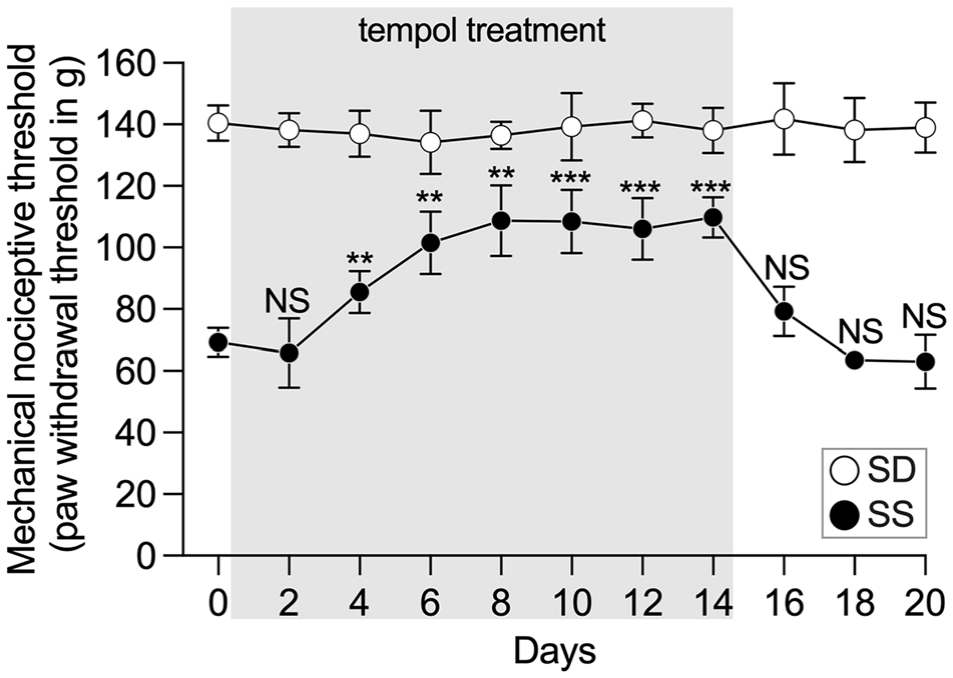
Participation of oxidative stress in the SS phenotype. SS (full symbols) and SD (empty symbols) rats were treated with the antioxidant tempol (10 nM) dissolved in the drinking water for 14 consecutive days. Mechanical nociceptive thresholds were evaluated by the Randall-Selitto test every other day until the 20th day. While tempol treatment did not impact the nociceptive threshold in the SD group (F_(3.885, 27.19)_ = 0.4894, *p* = 0.7384 when the nociceptive threshold over time was compared to the baseline, one-way repeated measures ANOVA followed by Bonferroni posttest), it produced a robust change in the SS group (F_(3.724, 26.07)_ = 29.63, *p* < 0.0001), significant after the 4th day of treatment (**p = 0.0003), that persisted until the 14th day (***p = 0.0002 when compared to the baseline). After the treatment was interrupted, the SS nociceptive thresholds returned to levels not significantly different from the baseline by day 16 (*p* = 0.3833, NS). n = 8 per group.

Increased oxidative stress contributes to chronic inflammation^32^, and elevated reactive oxygen species (ROS) also contribute to the pathophysiology of chronic pain^20^. SS rats have a known mutation in the enzyme nicotinamide adenine dinucleotide phosphate (NADPH) oxidase Nox2 subunit phox67^33^, which leads to increased ROS levels^16,34^. We therefore sought to determine whether oxidative stress contributes to the SS hyperalgesic phenotype. SS rats received the membrane-permeable superoxide dismutase (SOD) mimetic 4-hydroxy-2,2,6,6-tetramethylpiperidinoxyl (tempol, 10 mM), dissolved in the drinking water, for 14 days, which produced a significant increase in the hindpaw mechanical nociceptive threshold during the treatment (Fig. 4, *p* < 0.0001). Four days after the treatment was interrupted, the mechanical nociceptive thresholds returned to baseline levels (*p* > 0.9999, when the thresholds on the 4th day post-treatment were compared to baseline). This result suggests that ongoing oxidative stress contributes to the hyperalgesic phenotype in SS rats.

### The HPA axis is dysfunctional in SS rats

The HPA axis is the archetypal endocrine stress system, with an end product of cortisol in humans and corticosterone in rats. These glucocorticoids exert powerful immunologic and anti-inflammatory effects—restraining and/or terminating the innate immune response in the central nervous system by inhibiting nuclear factor kappa B (NF-κB)^35^. This in turn decreases the production and secretion of proinflammatory cytokines such as IL-1β and IL-18^36^. We therefore investigated whether SS rats demonstrate a hypo-corticosterone state that might also contribute to the proinflammatory environment and consequent hyperalgesia.

**Figure 5:**
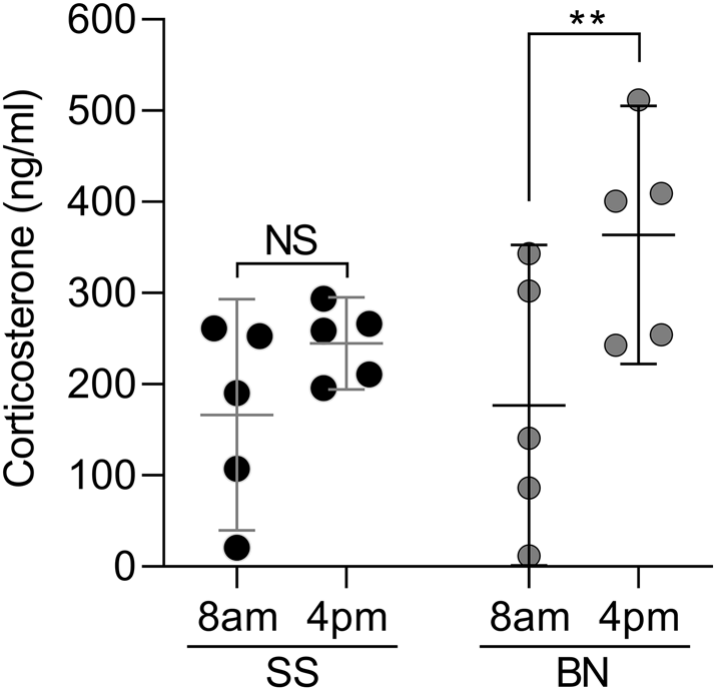
SS rats exhibit lower plasma diurnal corticosterone levels. To determine variations during the day in the levels of HPA activity, plasma corticosterone was measured in unstressed SS and BN rats at 8 am and 4 pm. Although corticosterone increased over the day in the BN group (t_4_ = 5.171, **p = 0.0066 when the 4 pm levels were compared to 8 am, paired Student’s t-test), in the SS, there was no significant variation (t_4_ = 1.708, *p* = 0.1628, NS), indicating a flattened diurnal rhythm. n = 5 per group.

Diurnal plasma corticosterone levels were measured in SS and BN rats^37,38^. As expected, the corticosterone levels were higher at the diurnal peak in BN rats (4 pm) compared to the diurnal nadir (8 am) (Fig. 5, *p* = 0.0066), reflecting its circadian rhythm. However, no AM-PM difference was observed in SS rats (*p* = 0.1628), suggesting a “flattening” of the corticosterone circadian rhythm. Of note, this lack of diurnal variation in corticosterone levels indicates that SS rats exhibit a centrally mediated decrease in peak HPA axis activity.

### SS rats exhibit impaired endogenous pain modulation

**Figure 6:**
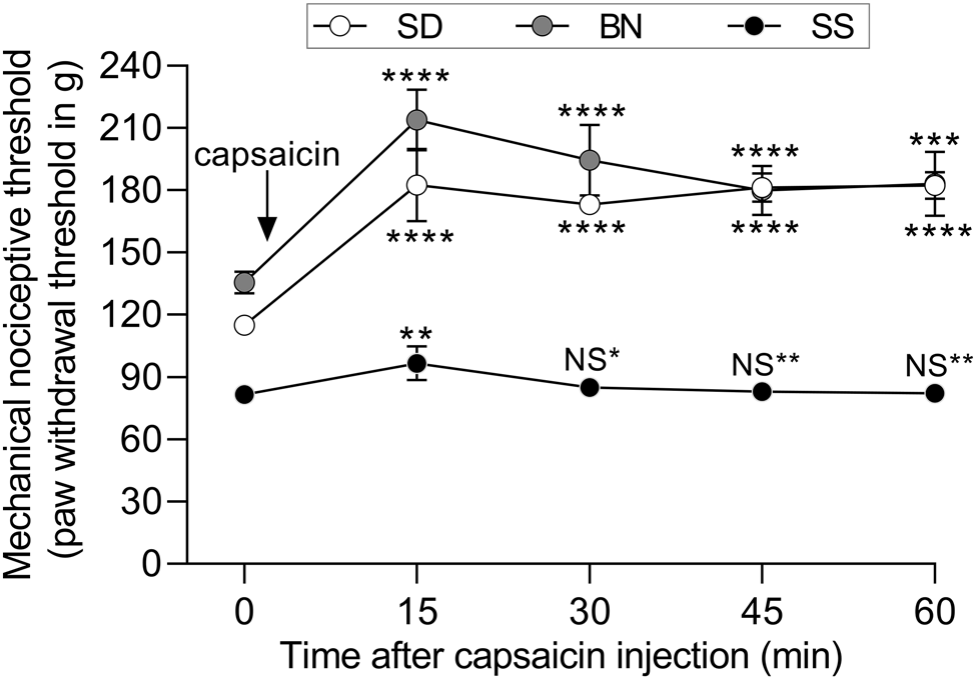
DNIC is impaired in SS rats. The baseline hindpaw withdrawal threshold in SS, SD, and BN rats was determined by the Randall-Selitto test. In sequence, a subdermal injection of capsaicin (125 µg, 50 µl) was performed into the right forepaw, followed by evaluation of the hindpaw withdrawal thresholds at 15 min intervals. While the hindpaw withdrawal threshold markedly increased in the SD (F_(1.825, 20.08)_ = 53.23, ****p < 0.0001, when the paw-withdrawal thresholds were compared to baseline, pre-capsaicin, thresholds, one-way repeated measures ANOVA followed by Bonferroni posttest) and BN (F_(3.139, 28.25)_ = 32.20, ****p < 0.0001 and ***p = 0.0002) rats, compatible with the presence of DNIC, in the SS group, no significant change in the nociceptive threshold was observed (F_(2.508, 62.71)_ = 7.772, **p = 0.0060, NS* *p* = 0.5308, NS** *p* > 0.9999), indicating that these rats did not exhibit DNIC responses. SD, n = 12; BN, n = 10; SS, n = 26.

Termed “conditioned pain modulation” in humans, diffuse noxious inhibitory control (DNIC) plays a key role in the central endogenous analgesia system and is a phenomenon whereby application of a strong nociceptive stimulus to one part of the body inhibits pain in multiple remote body regions^39^. DNIC is frequently described as an important regulator of pain signaling^40^ and defects in its mechanism have been suggested as a contributing factor to the pathogenesis of persistent and/or widespread pain conditions^21^. Thus, we evaluated the efficiency of DNIC in SS rats to determine whether this strain has impaired central, endogenous pain modulation mechanisms. Capsaicin (125 μg) was injected subdermally into the right front paw of the rats, and the mechanical paw-withdrawal thresholds were evaluated in the hindpaw every 15 min over an hour. While the nociceptive thresholds in SD and BN rats significantly increased after the remote application of the noxious stimulus, in the SS rats it was *absent* (Fig. 6, *p* < 0.0001 when the change in mechanical nociceptive threshold in SD and BN rats after capsaicin injection is compared to that in SS), indicating a dysfunction in the endogenous mechanism of pain control in SS rats.

### SS peripheral sensory neurons are hyperresponsive to proalgesic mediators

**Figure 7:**
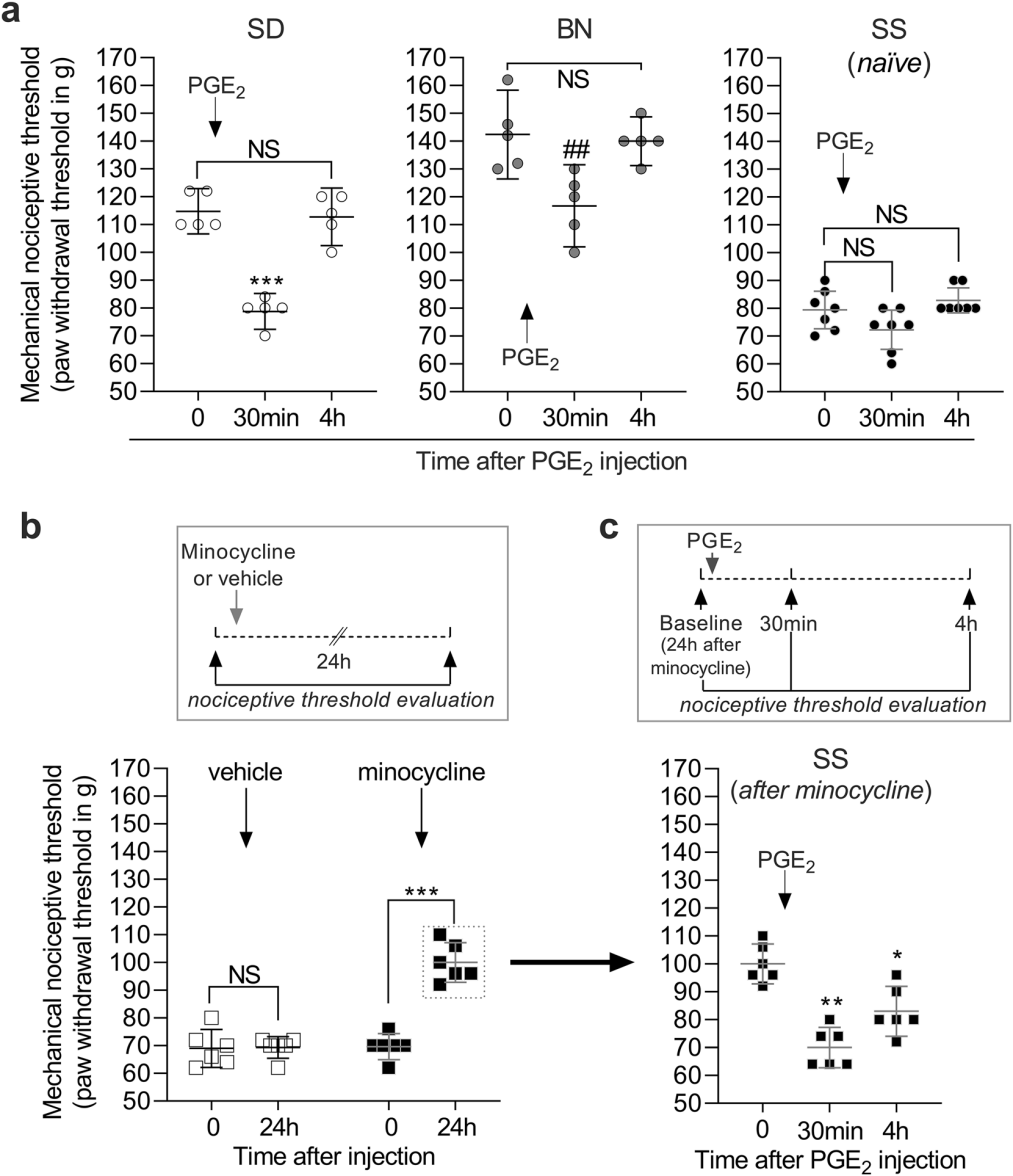
SS rats present increased responsiveness to the proalgesic mediator PGE_2_. (**a**): PGE_2_ (100 ng) was injected i.d. on the dorsum of the hindpaw of naïve SD (left), BN (middle) and SS (right) rats. Nociceptive thresholds were evaluated, by the Randall-Selitto test, before, 30 min and 4 h after injection. Significant decrease in threshold (hyperalgesia) was observed 30 min after PGE_2_ in both SD and BN rats (F_(1.471, 5.885)_ = 54.34, ***p = 0.0003 for SD; F_(1.178, 4.714)_ = 23.17, ^##^*p* = 0.0011 for BN, when compared to baseline, one-way repeated measures ANOVA followed by Bonferroni posttest) and, by the 4th h, the thresholds were not significantly different from the baseline (*p* > 0.9999 for both strains, NS). In the naïve SS group, however, the PGE_2_ effect was not evident due to the low nociceptive baseline threshold, close to the detection limits of the Randall-Selitto device (F_(1.339, 8.032)_ = 4.179, *p* = 0.3774 and *p* = 0.4616, both NS, when the thresholds at 30 min and 4 h are compared to pre-PGE_2_ levels, respectively). Thus, to visualize the PGE_2_ effect in SS rats, a separate group received minocycline ((**b**), full symbols, 30 mg/kg, i.p.). A control group (empty symbols) received vehicle. Evaluation of the nociceptive thresholds on the dorsum of the hindpaw 24 h later showed a significant increase in threshold in the minocycline-treated group (t_5_ = 8.113, ***p = 0.0005, when the thresholds before and after minocycline were compared, paired Student’s t-test; for the vehicle-treated group, t_5_ = 0.1465, *p* = 0.8893, NS). In sequence (24 h after minocycline), the minocycline-treated rats received an i.d. injection of PGE_2_ (100 ng) on the dorsum of the hindpaw (**c**), and the nociceptive thresholds were evaluated 30 min and 4 h later. A robust decrease in nociceptive threshold (hyperalgesia) was observed 30 min after PGE_2_ (F_(1.434, 7.171)_ = 27.75, **p = 0.0026, when compared to pre-PGE_2_ levels) that was still significant at the 4th h (*p = 0.0291), indicating a prolonged nociceptor sensitization. The schematics on the top of (**b** and **c**) describe the respective protocols used. This finding indicates that nociceptors in SS rats are hyperresponsive to proalgesic agents such as PGE_2_. Panel (**a**), SD, n = 5; BN, n = 5; naïve SS, n = 7; Panel (**b**) n= 6 for both groups; Panel (**c**), n = 6.

Plastic changes in peripheral sensory neurons have been observed in widespread pain conditions such as fibromyalgia and have been shown to contribute to pain severity^41,42^. A consequence of this neuroplasticity is an increased responsivity to pro-algesic mediators, which produces an exaggerated nociceptive reaction compared with unstimulated nociceptors^43^. We therefore evaluated the response produced by intradermal injection of prostaglandin E_2_ (PGE_2_) on the dorsum of the hindpaw to determine if increased nociceptor responsivity to inflammatory/hyperalgesic agents is also part of the SS phenotype. The effect of PGE_2_ on the mechanical nociceptive threshold is typically short when injected into the paws of SD rats, with a peak effect at 30 min and a duration of ~ 2 h, after which the threshold returns to baseline levels^44^. SS rats exhibit nociceptive thresholds close to the detection limits of the Randall-Selitto device, making it difficult to determine changes in sensitivity (Fig. 7a, panel on the right). We therefore treated SS rats with minocycline prior to injecting PGE_2_, to raise the nociceptive threshold (as shown in Figs. 3c and 7b) to a level that would allow us to identify the presence of nociceptive sensitization. After 24 h, we injected PGE_2_ (100 ng, i.d.) on the dorsum of the hindpaw of the minocycline-treated SS group and measured the nociceptive responses up to 4 h post injection (Fig. 7c). We observed that PGE_2_–induced decrease in mechanical nociceptive threshold was still present in those rats at the 4th h (*p* < 0.0291, when compared to baseline nociceptive threshold). However, when injected in naïve SD or BN rats (Fig. 7a, left and middle panels, respectively), the PGE_2_ effect was no longer present at that time point (*p* > 0.9999 for both strains). This finding indicated that the nociceptor response to proalgesic mediators is exacerbated in SS rats.

### Impact of pharmacological treatments on the SS hyperalgesic phenotype

**Figure 8:**
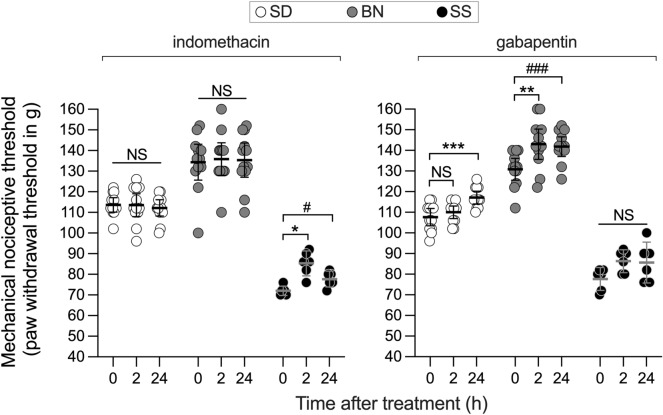
Effect of pharmacological treatments on the SS hyperalgesic phenotype. SS (closed symbols), SD (open symbols) and BN (gray symbols) rats were evaluated in the Randall-Selitto test to determine their baseline hindpaw withdrawal threshold. In sequence, a single injection with the NSAID indomethacin (left panel, 2 mg/kg, s.c.) or gabapentin (right panel, 100 mg/kg, i.p.) was performed. Mechanical nociceptive thresholds were evaluated 2 h and 24 h later. We found that the effect of either indomethacin or gabapentin was significantly different across the strains (F_(2, 27)_ = 95.81; F_(2, 27)_ = 207.0, respectively, *p* < 0.0001 for both, two-way repeated measures ANOVA followed by Bonferroni posttest). Left panel: While injection of indomethacin produced a small but significant increase in the mechanical nociceptive threshold in the SS rats, when evaluated 2 h post-injection (*p = 0.0140 compared to baseline) that was still significant 24 h later (#*p* = 0.0158), no significant effect was observed on SD or BN rats (*p* > 0.9999, NS, for both). Right panel: In contrast, treatment with gabapentin affected both SD (***p = 0.0004) and BN (**p = 0.0101; ###*p* = 0.0091) rats. However, no significant change in the withdrawal threshold was observed in the SS group (*p* > 0.9999). SS, n = 6; SD and BN, n = 12.

Finally, we tested two common classes of analgesics to determine their efficacy in treating the SS rat hyperalgesic phenotype. Since dexamethasone produced a significant attenuation of the nociceptive sensitivity in SS rats (Fig. 2a), indicating a role for inflammation, we tested the analgesic effect of the NSAID indomethacin in SS rats compared with SD and BN rats. Indomethacin was administered systemically (2 mg/kg, s.c.), and, the mechanical nociceptive threshold, evaluated on the hindpaw, 2 h and 24 h later. While no effect was observed in the SD and BN strains (*p* > 0.9999, for both), there was a significant increase in the nociceptive threshold in the SS group (*p* = 0.0140 at the 2 h time point, and *p* = 0.0158, 2 h later when compared to baseline). This finding is in agreement with the effect produced by dexamethasone and confirms the contribution of inflammation in SS hyperalgesia (Fig. 8, left panel). We have also evaluated the effect of the anticonvulsant gabapentin, used to attenuate painful conditions not associated with inflammation^45,46^, such as neuropathic pain. Systemic injection of gabapentin (100 mg/kg, i.p.) produced significant analgesia in the SD (*p* = 0.0004 when compared to baseline 24 h after injection) and BN (*p* = 0.0101 at 2 h and *p* = 0.0091 24 h after injection) strains, without affecting the SS rats (*p* > 0.9999) (Fig. 8, right panel). Together, these results suggest that the phenotype in SS rats does not involve a neuropathic component.

## Discussion

In the present study, we show that SS rats are a model of inherited hyperalgesia, as they exhibit mechanical pressure sensitivity in both paw and muscle without an external precipitating intervention. Accompanying the decrease in mechanical thresholds, SS rats show increased spontaneous pain behaviors induced by formalin, and also fail to mount a DNIC response to painful stimuli. This lack of central endogenous pain modulation suggests that the diminished mechanical thresholds do not just represent a trivial strain variation but may represent a hypernociceptive state. We also show that this strain demonstrates additional phenotypes consistent with chronic widespread pain conditions, such as dysfunction in stress response systems, neural inflammation and increased sensitivity to hyperalgesic mediators. Finally, treatment with dexamethasone, the reactive oxygen species scavenger tempol, or the glial inhibitor minocycline attenuated the pain sensitivity in SS rats without affecting the other strains, while indomethacin and gabapentin failed to show clinically significant pain relief. SS rats may be able to fill a face validity gap in current pain models because they exhibit inherited, idiopathic (as seen in a large percentage of patients with chronic pain), truly persistent, widespread pain.

Current models of widespread pain are produced by exposing rodents to difficult-to-adapt stressors, including repeated cold stress, unpredictable sound stress and sleep deprivation^47^. In addition, repeated administration of reserpine in rats causes long-lasting widespread muscle and cutaneous hyperalgesia that is sustained for 3 weeks^48,49^. Until now, the only reported inherited pain model of any kind has been the spontaneous trigeminal allodynia rat model^50^. Thus, SS rats are the first model of spontaneous widespread pain, as they exhibit bilateral hind limb mechanical pressure nociceptive sensitivity in both paw and muscle without an external precipitating intervention. The hypothesis that reduced mechanical thresholds in SS rats represent true hyperalgesia is supported by fMRI studies, which show significantly less functional connectivity in the brain pain inhibitory network (in areas such as the insula, periaqueductal gray, and cingular cortex) following noxious electrical stimulation in both FMS patients^51,52^ and SS rats compared with BN controls^53^.

In 1962, Dahl et al. selectively bred two contrasting lines of SD rats—*outbred* salt-sensitive (DS) and salt-resistant (DR) rats for the study of salt-induced hypertension^12^. In 1985, Rapp and Denne used these rats to derive Dahl/Rapp *inbred* salt-sensitive (SS/Jr) and salt-resistant (SR/Jr) strains, which were later distributed by Harlan, Moellegaard, Seiwa and other commercial breeders^54^. SS rats are used in experiments exploring the mechanisms causing salt-induced hypertension as they become profoundly hypertensive when fed a diet with 2–8% salt^55^. However, little is known about their neurologic phenotypes, including those associated with the nociceptive system. Only three previous studies have explored pain in the SS rat, and they present conflicting results: Taylor et al. showed that formalin-induced pain was independent of blood pressure^56^, while Friedman et al. reported hypertensive DS rats had reduced sensitivity to painful stimulation compared with normotensive controls^57^. The differences are likely due to the divergent genetic backgrounds of the strains used. Friedman used the *outbred* rats developed by Dahl, while the more recent Taylor study used the *inbred* rats developed by Rapp. The only study exploring pain phenotypes in SS/MCW rats on a normal salt diet is by Young et al.^58^, which compared the response of chronic constriction injury (CCI) in SS/MCW, BN, SD and fawn hooded rats, and found much higher pain sensitivity in SS/MCW, an observation consistent with the present study. This highlights the importance of the source of the SS rats, as the phenotypes are *inextricably* linked to the specific genetic background. It also suggests that the genes responsible for the pain phenotypes and those responsible for hypertension are unrelated; otherwise, the different strains should have had matching blood pressure and pain responses.

We used SS/MCW rats maintained at the Medical College of Wisconsin (MCW) since 1991 for all of our experiments. This strain originated from an inbred congenic control group of rats originally derived from the Harlan SS/Jr colony. It has since undergone considerable marker-selected breeding to eliminate residual heterozygosity and genetic contamination. To confirm homozygosity, the strain was tested with 200 microsatellite markers (genome-wide scan at 20 cM), all of which were homozygous for all regions tested^59^. MCW has made these rats commercially available through Charles River Laboratories. Unlike the original SS/Jr rat, this strain exhibits little or no change in blood pressure with age when fed a normal salt diet^55^. It is thought that the SS/MCW strain was contaminated with Lewis and SR/Jr DNA prior to inbreeding, which fixed the genetics^60^.

Patients with chronic renal failure often experience widespread pain. It would be tempting to attribute the pain symptoms seen in SS/MCW rats to a cardiovascular or renal cause. However, their renal function is quite well preserved. Even after 3 weeks of high salt, which produces profound hypertension and renal injury, their glomerular filtration rate (GFR) is only reduced by 28%^13^, placing them at the equivalent of stage 2 kidney disease (signs of kidney damage are present but eGFR is “normal”, GFR 89–60%)^61,62^. On a 0**.**4% sodium diet, such as that used in the current study, SS/MCW rats remain normotensive, have GFRs indistinguishable from control strains^13^ and never develop changes in cardiac size or function^14,15^. Therefore, it is unlikely that renal failure is the mechanism responsible for SS/MCW persistent widespread pain when fed a normal salt diet.

Instead, it is likely due to a currently unknown gene or set of genes. To date, mutations in the Cyp11b1, Adamts16, Rffl, Nlr2f2, Tmeff2, renin and NcF2 genes of SS rats have been identified as quantitative blood pressure trait genes of interest in salt-induced hypertension^33,63,64^. No studies have yet been conducted looking for alleles that influence pain phenotypes in SS/MCW rats. The SS/MCW rat is not a single gene mutation model. We view this as an advantage because it mirrors the genetic complexity of chronic widespread pain in humans. There has been an inability to confirm genetic associations in FMS candidate gene studies and a failure to replicate single nucleotide polymorphism (SNP) associations in independent cohorts^65^. Therefore, one should not expect that an animal model with a single gene mutation would replicate the complexity of FMS or other widespread pain conditions in humans. The SS/MCW rat is highly inbred and has been genetically sequenced and characterized at great depth (see Rat Genome Database, http://rgd.mcw.edu). For these reasons, we propose that the SS/MCW rat replicates both the phenotypic and genetic complexity of persistent widespread pain conditions. This provides an opportunity to explore the metabolic, physiological and genetic origins of widespread pain that other models do not provide.

We do not yet understand the complex interplay among environmental and genetic factors that lead to chronic pain. However, neural, endocrine and immune mechanisms probably play key roles^66,67^. In this study, we found that IL-1α and IL-18 were elevated in both the plasma and CSF of SS rats compared with BN and SD rats. Information regarding the function of IL-1α is limited and conflicting. Some studies report it as an anti-inflammatory cytokine^68^ that can mediate astrocyte protective signals in models of neuropathic pain^69^, while others show that it binds the same receptor as IL-1β, inducing the same proinflammatory effects^70^, and its contribution to pain associated with lumbar disk herniation^22^ and joint injury^71^. In the present study, we did not find significant levels of IL-1β and therefore attributed the antinociceptive effect of IL-1RA to competitive antagonism of IL-1α. In contrast, the proinflammatory function of IL-18 has been well characterized, with elegant studies demonstrating its contribution to neuropathic and bone cancer pain by regulating glial function^23,72^. We also found that the chemokine GM-CSF was elevated in the CSF but not the plasma of SS rats. GM-CSF is a growth factor for cells of the myeloid lineage, and the signal transduction induced by GM-CSF in these cells has been extensively studied^73^. Since GM-CSF produces numerous effects on microglia, including proliferation and morphologic changes, we measured glial staining in the PAG, concluding that neuroinflammation may be highly significant in SS rats. We therefore systemically administered the anti-inflammatory drug minocycline, which effectively reduced the SS hyperalgesic phenotype. Minocycline was selected for our initial screening as the microglial inhibitor most frequently used in the preclinical literature^74^. However, one significant limitation is that minocycline lacks selectivity; a recent critical review of minocycline as a microglial modulator clearly outlines off-target effects in peripheral immune cells and even neurons^75^. Future studies will be needed using a more specific inhibitor to fully delineate the role glial cells play in the SS rat hyperalgesic phenotype.

Increased oxidative stress can produce chronic inflammation^32^, which contributes to the pathophysiology of chronic pain^20^. ROS production begins with the generation of superoxide by NADPH oxidase, xanthine oxidase, nitric oxide synthase, cyclooxygenase, and components of the mitochondrial electron transport chain^20^. However, NADPH oxidase appears to be the primary source of excessive immune cell production of superoxide and can be generated by various NADPH oxidase isoforms, including Nox1–5, Duox1, and Duox2^76^. Notably, Nox2-deficient mice demonstrate reduced spinal ROS production and attenuated mechanical and thermal hypersensitivity after peripheral nerve injury. Moreover, the expression of microglial activation markers CD11b and Iba1, as well as the production of proinflammatory cytokines such as IL-1β, show significant attenuation in Nox2-deficient mice^77^. These findings suggest that superoxide derived from Nox2 contributes to microglial activation after peripheral nerve injury. Here, we show that the superoxide dismutase mimetic tempol significantly increased paw withdrawal thresholds in SS rats (but not SD rats), attenuating hyperalgesia. Since SS rats have a known mutation in the Nox2 subunit phox67^33^, which leads to increased ROS levels^16,34^, NADPH oxidase-mediated ROS production may, therefore, be one of the causes of elevated nociceptive cytokine and chemokine production in SS rats.

Another possible cause is a hypoactive HPA axis. An underrecognized effect of cortisol insufficiency is muscle and joint pain^78^, with case studies reporting pain as the initial presenting symptom of adrenal insufficiency^79^. “Flattening” of the cortisol circadian rhythm is also seen in FMS, suggesting that low cortisol levels contribute to this pain condition^80^. However, the mechanisms by which suppressed cortisol contributes to pain in humans are just beginning to be understood. We observed that SS rats exhibited an attenuated afternoon concentration of plasma corticosterone, reflecting a decreased centrally driven circadian peak in HPA axis activity^37,38^. If chronically lower corticosterone levels contribute to hyperalgesia in SS rats, then supra-physiological replacement of glucocorticoid activity could treat it. This would explain the effect of the glucocorticoid receptor agonist dexamethasone—selected due to its potency and selectivity (little mineralocorticoid activity)—on the SS paw withdrawal threshold, which was significantly increased in SS rats after treatment, suggesting that lower glucocorticoid levels in SS rats significantly contribute to their persistent pain.

Chronic overlapping pain conditions such as FMS, temporomandibular disease, migraine, and irritable bowel syndrome all share a phenotype with *reduced* or *absent* DNIC^81–84^. When tested in the perioperative setting, reduced DNIC efficiency was found to be highly predictive of patients who developed chronic pain following thoracotomy and cesarean surgeries^85,86^. This suggests that altered DNIC might be relevant in the pathogenesis of these chronic pain conditions. Our tests showed that while BN and SD rats exhibited intact DNIC, it was *absent* in SS rats. This finding provides the opportunity to use SS rats to investigate the mechanisms responsible for alterations in DNIC.

Persistent pain conditions frequently have abnormalities in both central and peripheral nociceptive processing. The impaired DNIC in SS rats indicates a deficiency in central endogenous pain modulation. We therefore sought to determine if there was also evidence of peripheral dysfunction. Some believe that plasticity in the peripheral nervous system is responsible for the pain hypersensitivity that characterizes chronic pain disorders^87^, as indicated by lower mechanical nociceptive thresholds in the absence of any noxious stimulation. Higher responsivity to inflammatory mediators has been suggested as an additional feature of generalized pain syndromes such as FMS, irritable bowel syndrome and interstitial cystitis^87^. Thus, we investigated the possible contribution of nociceptor neuroplasticity to the SS hyperalgesic phenotype. We found that the response to the proalgesic mediator PGE_2_ was exacerbated in SS rats compared to the other strains, indicating that nociceptors in the SS rat are hyperresponsive to noxious stimulation, a condition compatible with the presence of widespread pain^87^.

Many clinical studies define a clinically effective analgesic as one providing a > 30% improvement in pain scores^88^. This standard of efficacy is not routinely used in preclinical studies where effectiveness is most often defined as a statistically significant difference. We observed that only minocycline reached the level of clinically significant analgesia, despite seeing an increase in nociceptive thresholds by indomethacin. This is in line with the reported efficacy of NSAIDs and gabapentin in the treatment of fibromyalgia, where both classes of drugs are prescribed but with no evidence to support the ability of these medications to treat fibromyalgia pain^89,90^.

In summary, we have presented a series of tests that establish the SS rat as a model of inherited, widespread, persistent pain. This strain may be useful in drug identification and development studies or for identifying biomarkers for chronic pain diagnosis and treatment. Nevertheless, further investigation is needed to define the specific widespread pain condition that SS rats most resemble, including whether SS rats exhibit additional symptoms consistent with fibromyalgia or if they are prone to developing chronic postoperative pain.

## Materials and methods

### Experimental animals

Male Dahl salt-sensitive (SS) and Brown Norway (BN) rats were purchased from the Medical College of Wisconsin, WI, while male Sprague Dawley (SD) rats were purchased from Charles River Laboratory, USA, for use in this study. Rats were housed 2–3 per cage at the University of Utah Animal Facilities and kept in a temperature-controlled environment on a 12:12 h light–dark cycle (lights on at 6:00 am, lights off at 6:00 pm) with ad libitum access to food and water. SS rats were fed a special diet [AIN-76A Purified Rodent Diet with 0.4% Sodium Chloride (Pelleted, Dyed Green), from Dyets Inc]; SD and BN received regular chow (Teklad Global Soy Protein-Free Extruded Rodent Diet 2920X, from Envigo). Behavioral tests were performed between 9:00 am and 5:00 pm. All animal procedures were reviewed and approved by the Institutional Animal Care and Use Committee (IACUC) of the University of Utah and conformed to the National Institutes of Health *Guidelines for the Care and Use of Laboratory Animals*. Age matched 12 week old animals were used only once and, after the experiments were completed, euthanized by carbon dioxide inhalation followed by cardiac puncture as recommended by the University guidelines. The experiments herein reported follow the recommendations in the *Animal Research: Reporting of *In Vivo* Experiments* (ARRIVE) guidelines. Every effort was made to minimize the number of animals used and their suffering.

### Drugs

The compounds used in this study were: the algesic agent capsaicin, the inflammatory agent λ-carrageenan plant mucopolysaccharide (CARR), the non-steroidal anti-inflammatory drug (NSAID) indomethacin, the glial activation inhibitor minocycline hydrochloride, and the hyperalgesic mediator prostaglandin E_2_ (PGE_2_), all from Sigma-Aldrich, the glucocorticoid dexamethasone (from Tocris), the anticonvulsant / analgesic gabapentin (from Medisca), the interleukin 1 receptor antagonist (IL-1RA), and the oxidative stress attenuator 4-hydroxy-2,2,6,6-tetramethylpiperidinoxyl (tempol), both from Cayman Chemical. Capsaicin, dissolved and diluted in absolute ethanol to a concentration of 2.5 µg/µl, was injected subdermally (50 µl) into the plantar surface of the right front paw; CARR, dissolved and diluted in 0.9% saline to a concentration of 1%, was injected i.d. (5 µl) on the dorsum of the hindpaw using a 27-gauge needle. Dexamethasone (1 mg/kg) and indomethacin (2 mg/kg) were dissolved in dimethyl sulfoxide (DMSO, Sigma-Aldrich) and injected s.c. in the nape of the rat’s neck; minocycline and gabapentin, dissolved and diluted in distilled water, were administered i.p.; Tempol was dissolved in the drinking water to a concentration of 10 mM. PGE_2_, prepared from a stock solution (1 mg/ml in ethanol) and diluted in saline (final concentration of ethanol < 1%), was administered i.d. on the dorsum of the hindpaw (5 µl) using a 30-gauge hypodermic needle adapted to a Hamilton syringe (Reno, NV). The doses and routes of administration were based on previous studies^44,45,91–99^.

### Mechanical nociceptive threshold evaluation

Two tests were used to assess pain behavior: withdrawal response to weight application on the dorsum of the hind paw or on the gastrocnemius muscle using the Randall-Selitto device (Ugo Basile Analgesymeter; Stoelting, Chicago, IL) and von Frey filament stimulation of the plantar surface of the hindpaw.

The Randall–Selitto paw withdrawal test applies a linearly increasing force to the dorsum of the rat hind paw^100^ or to the gastrocnemius muscle to determine the mechanical nociceptive threshold. Rats were placed in acrylic cylindrical restrainers with triangular ports through which the hind legs were free to extend 15 min before the experiments for acclimation. The nociceptive threshold was defined as the mean of 3 subsequent readings, converted to force in grams, at which the rat presented either a flinching or withdrawal reaction of the paw or leg upon stimulation. Of note, to evaluate the muscle mechanical nociceptive threshold, the gastrocnemius area in the rat leg was shaved, and stimulation was performed with the probe inverted in its support, which allowed a larger area to be stimulated on the muscle area.

For the von Frey filaments tests, rats were placed in clear plastic chambers (20 × 10 × 13 cm) on a wire grid floor 15 min before the start of testing. The responses to stimulation were determined by applying the calibrated filaments from underneath the cage through the mesh floor grid to an area between the five distal footpads for up to 5 s, 3 times separated by a 10 s interval. If no response was elicited, the process was repeated with the next filament in caliber. A tilted mirror placed under the grid provided a clear view of the rat hindpaw, and the end point was characterized by the removal of the paw in a clear flinch response after paw withdrawal. Each filament caliber represented a specific force in grams, and the lowest force from 3 tests producing a reaction at least 2 out of the 3 stimulations was considered the nociceptive threshold.

### Inflammatory pain model

Inflammatory mechanical hyperalgesia was produced by i.d. injection of CARR (1%, 5 μL) on the dorsum of the hindpaw of SS, SD and BN rats as described above. Mechanical nociceptive thresholds were evaluated by a Randall-Selitto device before and 4 h after CARR administration.

### The formalin test

To evaluate the nociceptive behavior evoked by formalin, rats were placed individually in clear plastic chambers (20 × 10 × 13 cm) with a mirror positioned outside to facilitate the observation of the hindpaws. After a 15 min period of habituation to their surroundings, the animal was removed and gently restrained for s.c. injection of formalin (10% Buffered Formalin, from Fisher Scientific, diluted 10 times in saline to the final concentration of 1%), on the dorsum of the hindpaw (50 µl), using a 30-gauge needle. After returned to the chamber, the 90 min-observation period (divided into 12 blocks of 5 min) started. The test was divided in phases 1 (0–10 min) and 2 (15–90 min), as previously described^101,102^. The nociceptive behavior was determined by the number of flinches—or vigorous shaking—of the treated paw.

### Immunohistochemistry—Iba

Rats were transcardially perfused, under isoflurane anesthesia, with 1xPBS followed by neural buffered formalin, and had their brains harvested. The tissues were post-fixed in formalin overnight and sectioned at 40 microns using a Leica VT1200 S vibratome (Leica Microsystems Inc., Buffalo Grove, IL); serial sections were collected from the PAG area, then washed in 1xPBS and blocked with PBST (1xPBS with 0.2% triton x-100) containing 5% normal goat serum (Vector Laboratories). After blocking, the sections were incubated with rabbit anti-Iba1 antibody (1:500, Fujifilm Wako Chemicals, #019–19,741), diluted in PBST containing 3% NGS, overnight at 4 °C. The next day, they were washed 3 times for 5 min in PBST, followed by incubation with goat anti-rabbit secondary antibody (Alexa Fluor 568, 1:1000, Invitrogen #A-11036) for 2 h at room temperature. The sections were then washed 3 times for 10 min in PBS, mounted onto slides and sealed with mounting media containing DAPI (H-1200, Vector Laboratories). Images were taken using a Zeiss 700 confocal microscope, and the area of stain was calculated using the ImageJ software.

### Legendplex assay

Rats were anesthetized, and CSF and plasma samples were obtained using previously described methods^37,103^. Briefly, plasma samples were obtained via the tail clip method, while CSF collection was performed by direct cerebellomedullary cistern puncture of anesthetized rats using a collection apparatus with negative pressure. A bead-based immunoassay was used to quantify 13 rat cytokines simultaneously (LEGENDplex™ rat inflammatory panel, Biolegend). Reactions were performed in duplicate. Analysis was performed with a FACSCanto flow cytometer (BD Biosciences). Data were analyzed via Legendplex V8.0 software (Biolegend) and specified as pg/ml.

### Corticosterone sampling protocol

Each rat was handled daily for 5–10 min over at least 3 consecutive days prior to the experiment to ensure that they would be habituated to the examiner and would remain quiet and calm on the day of the sample collections. Following the days of habituation, when the animals were comfortable enough to curl up in the crick of the examiner’s elbow and try to fall asleep, each rat was removed from its cage and placed under a dark towel on a nearby table and gently restrained. Blood was sampled by tail clip (n = 5 per experimental group) for measurement of corticosterone concentration at 8 am and 4 pm to assess the baseline (unstressed) diurnal rhythm as described previously^38,97^. The tail was immersed in 42 °C water for 30 s before clipping the last 0.25 mm of the tail off to collect blood samples. Blood was collected in 0.5 ml EDTA lavender-topped collecting tubes from a lateral tail vein. The sample was immediately placed on ice, and within 10–15 min of collection, the lavender top tubes were centrifuged at 10,000 RPM at 4 °C for 10 min. The plasma supernatant was then carefully pipetted into labelled Eppendorf tubes and stored at − 80 °C. The plasma corticosterone concentration was measured by radioimmunoassay as previously described^38^.

### Statistical analysis

In all behavior experiments, the dependent variable was hindpaw withdrawal or gastrocnemius muscle reaction to mechanical stimulation, considered the nociceptive threshold. Data are presented as the mean with 95% confidence interval and expressed in grams, except for Fig. 1e, which expresses the percentage change in nociceptive threshold after CARR injection shown in Fig. 1d (with lower values representing higher pain sensitivity, and higher values representing lower sensitivity), and Fig. 1f, that shows the number of flinches produced by formalin injection. To determine the difference in baseline nociceptive threshold across the three strains (Figs. 1a and b), one-way analysis of variance (ANOVA) followed by Bonferroni posttest was performed. To compare the change in nociceptive threshold during development between the strains (Fig. 1c), two-way repeated measures ANOVA followed by Bonferroni posttest was used, which was also used to compare the effect of CARR injection, as shown in Fig. 1d, and the formalin-evoked nociceptive behavior (Fig. 1f), across the groups. The comparison of the effect of pharmacological treatments on the three strains was performed using two-way repeated measures ANOVA followed by Bonferroni posttest as well (minocycline in Fig. 3c, and indomethacin/gabapentin in Fig. 8). Paired Student’s t-test was used to determine the effect of drugs on the nociceptive threshold (CARR in Fig. 1d, minocycline in Fig. 7b) or the diurnal variation in corticosterone (Fig. 5) in individual groups. When the effect of treatments in two different groups was compared, unpaired Student’s t-test was used (Fig. 2a and c, for vehicle vs dexamethasone and vehicle vs IL-1RA, respectively). To determine the intensity of cell staining in the PAG in the immunohistochemistry images on Fig. 3, the differences in the concentration of cytokines or chemokines in the three rat strains (Fig. 2b), as well as to compare the mechanical nociceptive thresholds before, 30 min and 4 h after PGE_2_ injection in SS rats treated with minocycline (Fig. 7c), naïve SS rats, SD rats and BN rats (Fig. 7a), one-way ANOVA followed by Bonferroni posttest was used. Finally, the effect produced by a treatment in individual groups over time (tempol in Fig. 4 and capsaicin in Fig. 6) was analyzed by one-way repeated measures ANOVA followed by Bonferroni posttest. GraphPad Prism 8 (GraphPad Software, Inc., San Diego, CA) was used to plot the graphics and to perform statistical analysis; *p* < 0.05 was considered statistically significant.

## Data Availability

The datasets generated during and/or analyzed during the current study are available from the corresponding author on reasonable request.
